# Schizophrenia alters intra-network functional connectivity in the caudate for detecting speech under informational speech masking conditions

**DOI:** 10.1186/s12888-018-1675-1

**Published:** 2018-04-04

**Authors:** Yingjun Zheng, Chao Wu, Juanhua Li, Ruikeng Li, Hongjun Peng, Shenglin She, Yuping Ning, Liang Li

**Affiliations:** 1 The Affiliated Brain Hospital of Guangzhou Medical University (Guangzhou Huiai Hospital), Guangzhou, 510370 China; 2 Faculty of Psychology, Beijing Normal University, Beijing, 100875 China; 3 School of Psychological and Cognitive Sciences, Beijing Key Laboratory of Behavior and Mental Health, Key Laboratory on Machine Perception (Ministry of Education), Peking University, 5 Yiheyuan Road, Beijing, 100080 People’s Republic of China; 4 Beijing Institute for Brain Disorder, Capital Medical University, Beijing, China

**Keywords:** Schizophrenia, Speech detection, Precedence effect, Functional connectivity, Masking, Caudate

## Abstract

**Background:**

Speech recognition under noisy “cocktail-party” environments involves multiple perceptual/cognitive processes, including target detection, selective attention, irrelevant signal inhibition, sensory/working memory, and speech production. Compared to health listeners, people with schizophrenia are more vulnerable to masking stimuli and perform worse in speech recognition under speech-on-speech masking conditions. Although the schizophrenia-related speech-recognition impairment under “cocktail-party” conditions is associated with deficits of various perceptual/cognitive processes, it is crucial to know whether the brain substrates critically underlying speech detection against informational speech masking are impaired in people with schizophrenia.

**Methods:**

Using functional magnetic resonance imaging (fMRI), this study investigated differences between people with schizophrenia (*n* = 19, mean age = 33 ± 10 years) and their matched healthy controls (*n* = 15, mean age = 30 ± 9 years) in intra-network functional connectivity (FC) specifically associated with target-speech detection under speech-on-speech-masking conditions.

**Results:**

The target-speech detection performance under the speech-on-speech-masking condition in participants with schizophrenia was significantly worse than that in matched healthy participants (healthy controls). Moreover, in healthy controls, but not participants with schizophrenia, the strength of intra-network FC within the bilateral caudate was positively correlated with the speech-detection performance under the speech-masking conditions. Compared to controls, patients showed altered spatial activity pattern and decreased intra-network FC in the caudate.

**Conclusions:**

In people with schizophrenia, the declined speech-detection performance under speech-on-speech masking conditions is associated with reduced intra-caudate functional connectivity, which normally contributes to detecting target speech against speech masking via its functions of suppressing masking-speech signals.

## Background

Compared to healthy people, people with schizophrenia perform worse in recognizing speech under adverse listening conditions [1–7]. For example, both first-episode patients and chronic patients with schizophrenia perform worse than their matched healthy controls in recognizing target speech when a masker, particularly a two-talker-speech masker is presented [3]. Up to date, the brain substrates underlying the schizophrenia-related augmentation of the vulnerability of speech recognition against informational speech masking remain largely unknown.

Successful speech recognition under a speech-on-speech-masking condition involves multiple perceptual/cognitive processes, including target-speech detection, selective attention, sensory/working memory, and speech production. It is not surprising that speech recognition involves multiple brain regions with various perceptual/cognitive functions [5–10]. Although the augmented vulnerability to speech masking in people with schizophrenia may be associated with deficits of various perceptual/cognitive processes [5–7, 11–14], it is the most important of all to know whether deficits in speech detection (the early-stage process) are the primary cause leading to deficits of speech recognition against informational speech masking. We have recently reported that the performance in the task of target-speech detection, conducted by button-press, is poorer in people with schizophrenia than healthy listeners [7]. However, the underlying mechanisms have not been reported in the literature.

Interestingly, although people with schizophrenia perform worse in speech recognition under speech-on-speech masking conditions, they can still improve their speech recognition by using some perceptual/cognitive unmasking cues, such as auditory speech primes [5] and auditory precedence-effect-induced perceptual spatial separation (PSS) between target speech and masking speech [7]. Relative to the auditory precedence-effect-induced perceptual spatial co-location (PSC) listening condition, introducing the PSS condition can facilitate selective attention to the target speech [7, 15–17]. Note that relative to the PSC condition, the PSS condition does not substantially affect the signal-to-masker ratio in sound pressure level and the compactness of sound image when the two spatially separated loudspeakers are symmetrically placed relative to the listener [15]. However, it has not been reported in the literature whether speech detection under speech-on-speech masking conditions can also be improved by perceived spatial separation that is induced by the auditory precedence effect.

Moreover, both intra-network functional connectivity and inter-network functional connectivity essentially underlie information processing in the brain [15, 18–23]. Increased intra-network connectivity reflects regional increases in the strength of functional integration within the network [19, 24]. When confronted with changing cognitive demands, the human brain shows its ability to reconfigure network organizations selectively and adaptively to achieve an optimal balance between segregation and integration [19]. People with “precocious” expression of the within-network connectivity profile during early development exhibit superior cognitive functioning [23]. Also, disconnection of specific brain network modular have been found to be related to cognitive dysfunction or mental disorders [25–28]. For example, increased intra-network connectivity between particular DMN regions is associated with the severity of positive symptoms in patients with schizophrenia, suggesting a link between disorganized DMN and psychosis [24, 29, 30]. Also, decreases in functional connectivity (FC) within the social-cognitive network predicts the severity of deficits in impoverished speech and flattened affect in patients with schizophrenia [25]. It has been suggested that abnormities in intrinsic within-networks in patients with schizophrenia and their first-degree relatives indicate potential psychosis endophenotypes [26, 30]. Previously, we have reported that relative to the masker-only condition, patients with schizophrenia showed reduced BOLD activation in the regions of the superior parietal, precuneus, left mid-cingulate, and left caudate under the PSS listening condition [7]. So far, it is not clear whether the brain regions, whose intra-network functional connectivity are underlying speech detection against informational masker, are impaired in people with schizophrenia.

Using the functional resonance magnetic imaging (fMRI) methods, this study aimed to explore differences specifically in intra-network FC for detecting target speech against speech masking between listeners with schizophrenia and healthy listeners by re-analyzing part of data obtained from the Zheng et al. study (2015), whose main focus was to investigate the difference in the unmasking effect (the release of target speech from informational masking) between participants with schizophrenia and healthy controls [7]. First, the networks underlying speech-detection-task were identified for participants using the group independent component analysis (ICA) [31, 32]. Second, to detect the group difference in spatial pattern of each network, each component (network) estimated from ICA were compared between listeners with schizophrenia and healthy controls (with sex, age, educational level, and head-motion parameters as nuisance covariates). Next, mean FC within each schizophrenia-altered network (i.e., intra-network FC) was calculated and normalized with Fisher r-to-z transformation for each participant. Last, partial correlation was used to explore the relationship between the target-speech detection against speech masking and the intra-network FC of each schizophrenia-altered network in patients with schizophrenia (with sex, age, educational level, head-motion parameters, severity of psychotic symptoms, ill-duration, and dosage of antipsychotics as covariates) and healthy controls (with sex, age, educational level, and head-motion parameters as covariates) separately.

## Methods

### Participants

Participants with schizophrenia, diagnosed with the Structured Clinical Interview for DSM-IV (SCID-DSM-IV) [33], were recruited in the Affiliated Brain Hospital of Guangzhou Medical University (the Guangzhou Huiai Hospital) with the recruiting criteria used previously [5–7]. Exclusion criteria included comorbid diagnoses, alcoholic or drug abuses, histories of nervous or auditory system diseases, ages younger than 18 or older than 59 years, and/or other conditions that affected experimental tests (including a treatment of the electroconvulsive therapy (ECT) within the past three months or a treatment of trihexyphenidyl hydrochloride with a dose > 6 mg/day). Some of the patient participants received benzodiazepines based on doctors’ advice for the purpose of improving sleeping. All the participants used Mandarin Chinese as the first language. They were all clinically stable during their participation.

Healthy control participants were demographically matched to the patient participants. They were recruited from the communities around the hospital with the recruiting criteria used previously [29, 30, 33]. More in detail, these healthy participants were first telephone interviewed and then only those who passed the telephone interview were screened with the SCID-DSM-IV as used for patient participants. Each of the selected healthy controls had no history of Axis I psychiatric disorders as defined by the DSM-IV.

Both all the participants (including healthy controls and patient participants) and the guarantees of the patient participants gave their written informed consent for participation in this study. The Independent Ethics Committee (IEC) of the Guangzhou Brain Hospital approved the procedures of this study.

In total, 22 patient participants and 17 healthy controls participated in the study. However, 3 patient participants and 2 healthy controls were excluded from data analyses due to either excessive head movements (more than 3 mm in translation and/or 3°in rotation from the first volume in any axis) or failure to button-press responses during the fMRI scanning. Finally, 19 patient participants (8 females and 11 males) and 15 healthy controls (8 female and 7 males) were remained in fMRI data analyses. All the participants had normal pure-tone hearing at each ear (no more than 30 dB Hearing Level) at frequencies from 125 to 8000 Hz.

### Stimuli

The speech stimuli used in this study included target speech and masking speech. The target-speech stimuli were Chinese nonsense sentences. Each of the sentences contained 6 words and each word contained 2 syllables. These nonsense sentences were not semantically meaningful even though they were syntactically ordinary [1, 7, 34]. For example, the English translation of a Chinese nonsense sentence is “Those *directions* always *understand* my *gate*” (the keywords are italic). Clearly, the sentence frame cannot offer any contextual support for recognizing any individual keywords. Target speech was spoken by a young female talker (Talker A).

The speech masker was a 47-s loop of digitally-combined continuous recordings for Chinese nonsense sentences spoken by two other young female talkers (Talkers B and C). All the keywords in masking sentences did not appear in target sentences.

To produce virtual sound images that appeared to occur under free-field listening conditions, head-related transfer functions (HRTFs) were used to digitally process all the speech signals. The speech signals were filtered with the HRTFs to simulate source locations at 90-degree left and 90-degree right to a participant in the azimuth, respectively [7]. Based on both the HRTF and the precedence-effect paradigm, the target speech and masking speech were perceived as as being delivered by each of the two spatially separated “loudspeakers” in the frontal field. The inter-“loudspeaker” interval for both target speech and masking speech was 3 ms. More in detail, under the PSC listening condition, both the onset time of the target sound and that of the masker sound presented from the left headphone either led or lagged behind those from the right headphone by 3 ms. Due to the auditory precedence effect, participants perceived a fused target-speech “image” and a fused masking-speech “image” as coming from the same location. On the other hand, under the PSS listening condition, the onset time of the target sound presented from the left headphone led that from the right headphone by 3 ms, but the onset time of the masker sound presented from the left headphone lagged behind that from the right headphone by 3 ms. Also due to the auditory precedence effect, the perceptually fused target-speech image was perceived as coming from the left location and the perceptually fused masker-speech image was perceived as coming from the right location [7, 16, 35].

### Imaging equipment

A 3.0-Tesla Philips Achieva MRI scanner (Veenpluis 4–6, 5680 DA Best, Netherlands), which was set up in the Guangzhou Brain Hospital MRI Facility, was used to obtain blood-oxygen-level-dependent (BOLD) gradient echo-planar images (64 × 64 × 33 matrix with 3.44 × 3.44 × 4.6 mm^3^ spatial resolution, echo time = 30 ms, time to repeat = 9000 ms, acquisition time = 2000 ms, flip angle = 90, field of view = 211 × 211 mm^2^). High-resolution T1-weighted structural images (256 × 256 × 188 matrix with the spatial resolution of 1 × 1 × 1 mm^3^, repetition time = 8.2 ms, echo time = 3.8 ms, flip angle = 7°) were subsequently obtained.

Speech stimuli were delivered with a magnetic resonance-compatible pneumatic headphone system (SAMRTEC, Guangzhou, China) driven by Presentation software (Version 0.70). The target sound-pressure level was 90 dB SPL (before attenuation by earplugs) and the signal-to-masker ratio (SMR) was − 4 dB.

### Design and procedures

**Fig. 1:**
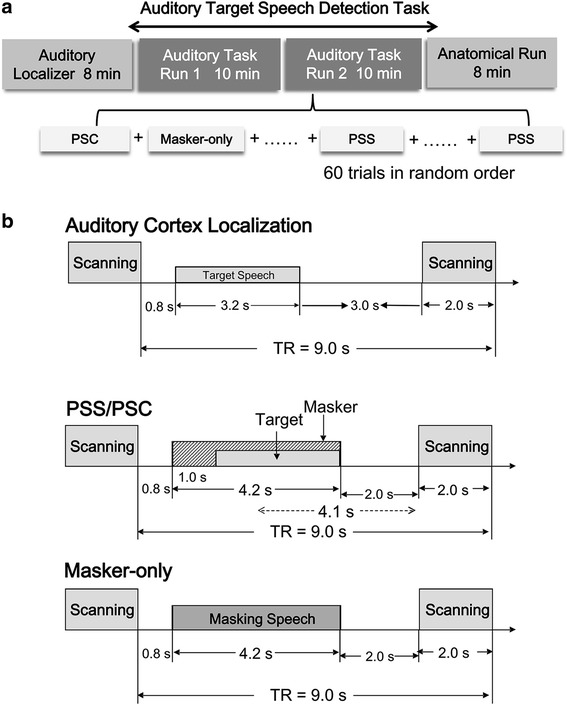
Illustrations of the fMRI experimental procedure. **a** Both the first experimental run and the second experimental run comprised 20 trials for each of the three listening conditions (PSS, PSC, and baseline) that were presented in random order for a participant. **b** The masking-speech and target-speech stimuli were presented 800 ms and 1800 ms after the end of the previous scanning, respectively. The target and the masker terminated at the same time. The midpoint of the auditory stimulus was presented 4.1 s prior to scanning. TR = Time to Repeat; TA = Acquisition Time

The whole scanning course contained an 8-min run for localization of the auditory cortex, an 8-min structure-scanning run, and two 10-min identical functional scanning runs. An event-related fMRI design was used for the functional run. In total 61 volumes were acquired from each participant over the first scanning run for the localization of the auditory cortex. The target speech with zero interaural time delay and the silence (rest) were presented alternately 500 ms after the scanning phase. Data of the first run were not included in the analysis. Sixty-one scanning trials were used for each functional run with a single dummy image obtained at the beginning (not included in data analyses) of each run and 60 experimental trials (20 trials for each of the three conditions: PSS, PSC, and baseline stimulation) (Fig. 1). The baseline-stimulation condition was the one that only the masking speech was presented. For an individual participant, the 60 trials across the 3 stimulation conditions were presented in a random order. For each participant across the two functional scanning runs, in total 120 volumes were acquired and included in data analyses. In each condition, 40 images were collected.

To avoid the effect of machine noise on image data collection, the sparse-imaging technique [36] was used: Speech stimuli were presented only during the silent period of the scanner between successive scans (Fig. 1). Also, to ensure that the hemodynamic responses evoked by the speech stimulus peaked within the scanning period, in each trial the midpoint of the speech stimulus was presented 4100 ms prior to the onset of the next scanning [36, 37].

In a scanning trial with either the PSC or PSS condition (Fig. 1), the speech masker was presented 800 ms after the last scanning trial. About 1 s later, the target sentence was presented. Then the target sentence terminated with the masker. In a scanning trial with the baseline-stimulation condition, only the masker sentence (without target-speech presentation) was presented 800 ms after the last scanning trial with a duration of 4200 ms.

Prior to scanning, all participants were screened for MR safety. To ensure that participants understood the instruction and knew how to conduct their button-press responses, a brief training was conducted. Speech sentences used in training were different from those in experimental scanning. The task of the participant inside the scanner was to detect the presence of the target speech against the masking speech. In a run, the ratio between the number of target-sound presence and the number of target-sound absence was 2:1 (e.i., 40 trials with the target-masker co-presentation and 20 trials with the masking-only presentation in random order). Participants were instructed to either press the left button on a response box using their right index finger if they detected the occurrence of a target sentence or press the right button if they did not. Participants’ responses were recorded and the hit rate (percentage of correct response) was calculated for each participant.

### fMRI data preprocessing

All fMRI data were processed and analyzed using the functional connectivity toolbox v17 (CONN, https://www.nitrc.org/projects/conn/) [38]. The pre-processing pipeline included participant motion estimation and correction, structural segmentation and normalization (re-sampling to a voxel size of 2 × 2 × 2 mm^3^ in the standard Montreal Neurological Institute (MNI) space), ART-based functional outlier detection and scrubbing, and functional spatial smoothing with an 8-mm Gaussian kernel. Before the first level analysis, the de-noising step (linear regression and band-pass filtering) was conducted to remove possible confounds including BOLD signal from the white matter and CSF, realignment parameters (6 motion parameters and 6 first-order temporal derivatives), and scrubbing parameters (maximum inter-scan movement and identified invalid scans) and task-design effects. The waveform of each brain voxel was filtered using a bandpass filter (f > 0.008) to reduce the effect of low-frequency drift [38].

### Independent component analyses (ICA)

Group ICA enables voxel-wise testing of the components images or fitting of a model to the component time-courses [32]. This process includes the following three steps [31, 32, 39, 40]: (1) reduction of the data dimensionality via principle component analysis (PCA), which includes optional subject-level dimensionality reduction, subject/condition concatenation of BOLD signal data along temporal dimension, and group-level dimensionality reduction to the target number of components, (2) application of the ICA algorithm to the data, and (3) back reconstruction for each individual participant.

After back-reconstruction, the IC time-courses and spatial maps for each participant and each condition (PSS, PSC and masker-only) were acquired. A minimum-description-length (MDL) algorithm [41] was used to determine the number of source locations. The average (integral) number of the components was 20, estimated across all participants. To identify the valid networks, the components were first examined visually to determine obvious artifacts, and then were correlated spatially to the templates (in SPM12) of probabilistic gray matter, white matter, and cerebrospinal fluid using multiple regressions. The components showing low associations (|β| <  0.5) with GM and high association (|β| > 2) with WM and CSF were considered as artifacts [26]. Next, the IC spatial pattern of each network (with the PSS condition and the PSC condition combined) was entered into a one-sample t test in SPM12 and the significance level for each network was adjusted for *p* < .0025 (voxel-wise family-wise-error [FWE] correction). Finally, the six components were regarded as noise, and the rest 14 ICs were considered for further analyses. The statistical maps were created with T-value larger than 15 (for the purpose of improving the representativeness of each component) (Fig. 3).

The difference in IC pattern within each network between participants with schizophrenia and healthy controls were compared using a two-sample t test in SPM 12, with age, sex, educational years and head-motion parameters (frame-wise displacement, FD) as nuisance covariates. A cluster-defining threshold (CDT) of with the *p* value of 0.001 and a cluster based FWE-corrected threshold with the *p* value of 0.05 was used to correct multiple comparisons.

### Intra-network functional connectivity

The intra-network (within-network) FC of a voxel was defined as the averaged FC (Pearson correlation) of that voxel to the rest of the voxels within the pre-defined network [18, 20]. First, the spatial map of certain component (network) estimated from ICA was used as the pre-defined mask. Then, the FC of each voxel to the rest of the voxels in the network (mask) was computed one-by-one and averaged as the Intra-network FC of this predefined network (averaged across the PSS and the PSC condition). Finally, individual-level FC was normalized using Fisher’s *z*-transformation. A two-sample *t*-test was used to compare group differences in the intra-network FC, with age, sex, educational years, and FD as covariates. Multiple comparisons were corrected using the Benjamini-Hochberg standard false-discovery-rate (FDR) method.

### Correlation analyses

Spearman correlation analyses were performed using SPSS 20.0 software to investigate the association between the strength of intra-network FC (Z-score) and the behavioral performance (percent-correct of target speech detection). Multiple comparisons were corrected using the Benjamini-Hochberg standard FDR method.

## Results

### Characteristics of participants

Between patient participants with schizophrenia and healthy controls, there was no difference in age, sex, educational years, or head-motion (FD) during scanning (all *p* values > 0.11). During this study patient participants received antipsychotic medications with the average chlorpromazine equivalent of 574 mg/day (based on the conversion factors described by Woods, [42]). On the day of fMRI scanning, the locally validated version of the Positive and Negative Syndrome Scale (PANSS) tests [43, 44] was conducted for all participants. Table 1 shows the characteristics of patient participants and those of healthy controls.

**Table 1:** Characteristics of Healthy Participants and Patients with Schizophrenia

	Patients	Healthy Participants	Statistics	
Characteristic	(n = 19)	(n = 15)	t/χ^2^	*p*
Age(years±SD)	33.05 (10.01)	30.13 (9.43)	0.54	0.591
Male% (n)	59.00 (11)	47.00 (7)	1.15	0.811
Education(years±SD)	12.89 (3.07)	14.73 (2.81)	1.67	0.105
MID (years±SD)	7.79 (6.56)	NA		
PANSS total	53.68 (5.66)	NA		
PANSS positive	15.21 (4.33)	NA		
PANSS negative	10.84 (4.00)	NA		
PANSS general	27.89 (3.89)	NA		
FD	0.19 (0.06)	0.16 (0.11)	0.78	0.443
Diagnostic subtype	N			
Paranoid	7			
Non-paranoid	12			
Medication				
typical	9			
atypical	16			
Typical/atypical*	6			
Chlorpromazine Equivalent	Mean:574.00 SD:346.34 Range:200–1600			

SD: Standard deviation; PANSS: Positive and Negative Syndrome Scale; MID: Mean illness duration; FD = frame-wise displacement; NA: not applicable; N = number of participants * Note that 6 patients received 2 different antipsychotic medications

### Performance of speech detection

**Fig. 2:**
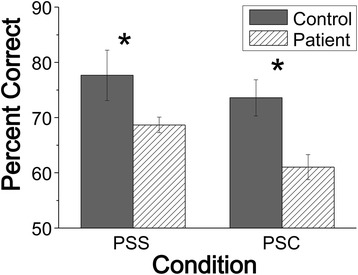
Percent correct of behavioral response in the target-speech detection task in patients with schizophrenia and healthy controls under either the PSS condition or the PSC condition. PSS = perceived spatial separation, PSS = perceived spatial co-location

Since the HRTF and precedence-effect procedures were applied, target-speech and masking-speech images were perceived as from either the same location (under the PSC condition) or different locations (under the PSS condition) in the frontal field. As Fig. 2 shows, the percent correct of button-press response in detecting target sentences was worse in patients than that in healthy controls under either the PSS condition or the PSC condition when the SMR was − 4 dB.

A 2 (group: control, patient) by 2 (spatial cue: PSS, PSC) ANOVA showed that the main effect of group was significant (F_1,66_ = 11.751, *p* = 0.001), the main effect of spatial condition was only marginally significant (F_1,66_ = 3.472, *p* <  0.067), and the interaction between group and spatial condition was not significant (F_1,66_ = 0.323, *p* = 0.571). Thus, the percent-correct of button-press response in detecting target sentences was significantly worse for patients than that for the healthy controls under either the PSS condition or the PSC condition. Also, the performance improvement in detecting target speech was not significant as the listening condition shifted from the PSC one to the PSS one.

### Networks of target-speech recognition against informational speech masking

**Fig. 3:**
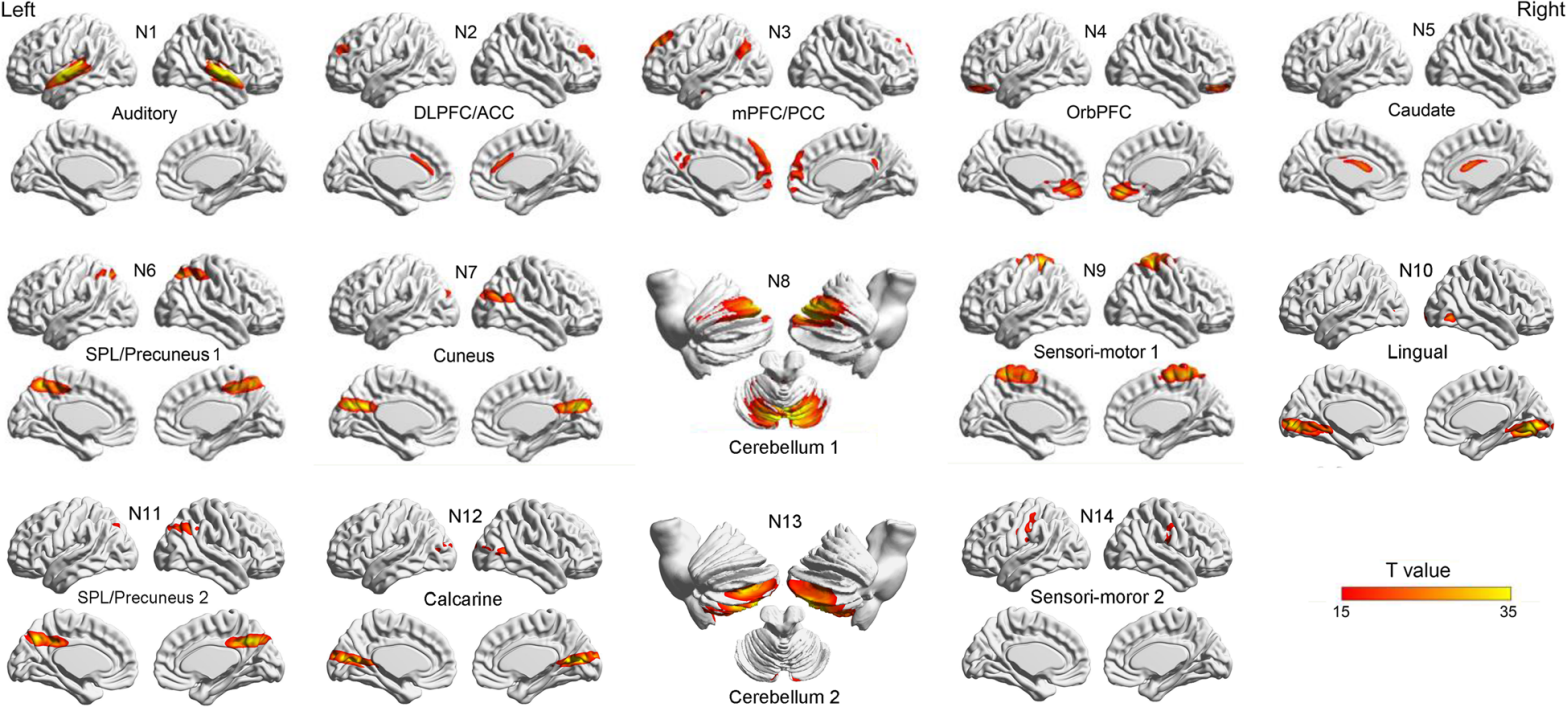
Cortical representations of the brain networks identified by independent component analyses (ICA). Fourteen of the meaningful and identifiable components were mapped to the template with a threshold of T larger than 15 (for the purpose of improving the representativeness of each component). DLPFC: dorsal lateral prefrontal cortex; mPFC: medial prefrontal cortex; OrbPFC: orbital prefrontal cortex; SPL: superior parietal lobule; PCC: posterior cingulate cortex. The map was visualized with the BrainNet Viewer (http://www.nitrc.org/projects/bnv/)

The task networks (Fig. 3), to some extent, were reconstructed compared to the resting-state networks described in previous studies [45–47]. The auditory network was composed of the bilateral STG (N1); the dorsal lateral prefrontal cortex (DLPFC) and anterior cingulate cortex (ACC) were coupled together (N2); the medial prefrontal cortex (mPFC) and the posterior cingulate cortex (PCC) constituted an independent network (N3); the sensory-motor network was composed of the bilateral precentral and postcentral cortex (N9 and N14); the Network N4 and N5 consisted of the bilateral orbital prefrontal cortex (OrbPFC) and bilateral Caudate, respectively. The N6 and N11 revealed two networks of bilateral precuneus and superior parietal lobule (SPL); the N8 and N13 were composed of two networks of the cerebellum; the N7, N10 and N12 consisted of bilateral cuneus, bilateral lingual and bilateral calcarine cortex, respectively (Fig. 3).

### Altered spatial activity pattern in the target-speech-detection network in patients with schizophrenia

**Fig. 4:**
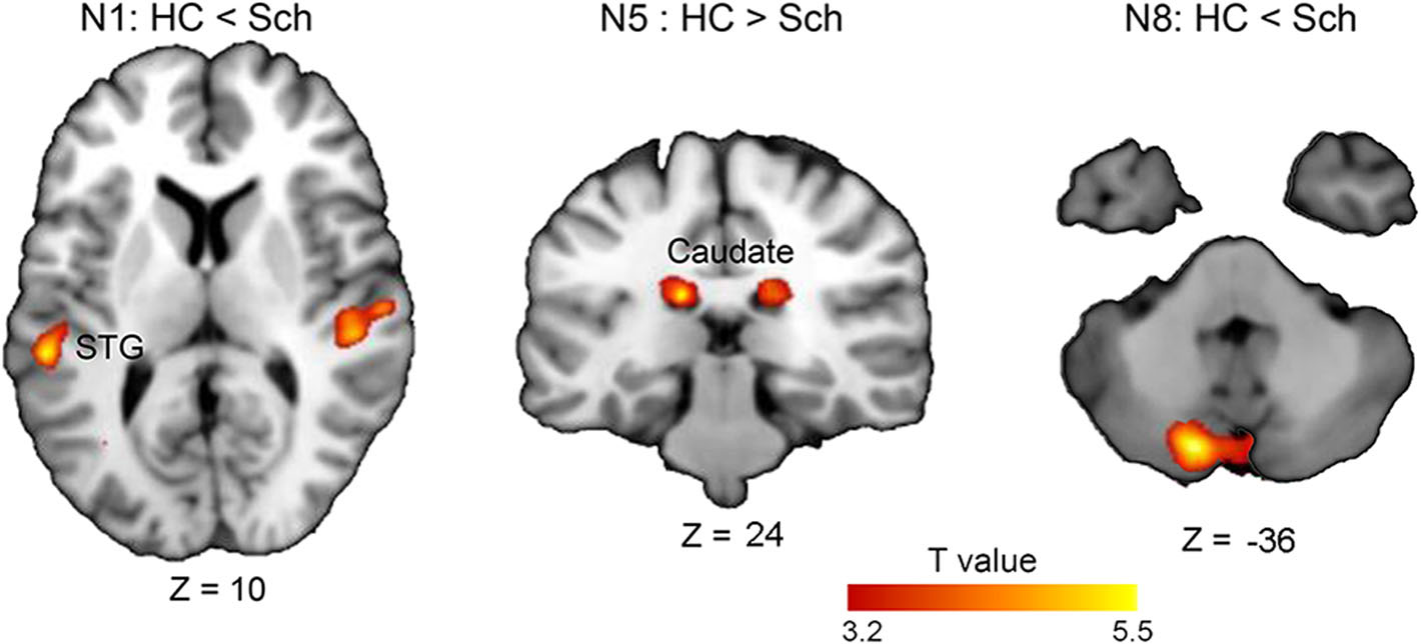
Components showing significant difference between the healthy controls (HC) and patients with schizophrenia (Sch) under the PSS and PSC conditions combined. A cluster-defining threshold (CDT) of *p* = 0.001 (*T* = 3.21) and a cluster based FWE–corrected threshold of *p* = 0.004 (for correction of multiple group comparisons) was used

To determine the critical brain networks that exhibited altered activity pattern in patients with schizophrenia, the fourteen components estimated from ICA (with the PSS condition and the PSC condition combined) were compared between patients with schizophrenia and healthy controls. The results showed that compared to healthy controls, patients showed significantly decreased covariation in the bilateral caudate, but significantly increased covariation in the cerebellum network (the left cerebellum) and the auditory network (bilateral STG) (Fig. 4 and Table 2, *p* < 0.05, cluster-wise FDR corrected). No significant difference in IC pattern was found between patients and controls under the baseline (masker-only) condition. Thus, compared to healthy controls, patient participants exhibited altered intra-network spatial covariation for the caudate, bilateral STG, and the cerebellum during target-speech detection task.

**Table 2:** Coordinates of the Brain Regions with Significant Difference in the Spatial Networks between the Healthy Controls and Patients with Schizophrenia with the Combination between the PSS Condition and the PSC Condition

Network	Contrast	Coordinates			Statistics				Location
		X	Y	Z	k	T	Z-score	*p*_*FWE*_	
N5	HC > Sch	−14	−26	26	168	5.48	5.00	0.003	L Caudate
		12	−24	22	123	5.26	4.83	0.005	R Caudate
N1	Sch > HC	−54	−32	10	362	5.41	4.93	0.003	L STG
		50	−22	−4	600	6.70	5.86	< 0.001	R STG
N2	Sch > HC	14	68	12	148	4.47	4.18	0.013^#^	L SFG
N8	Sch > HC	−12	− 76	−36	160	5.49	5.00	0.003	L Cerebellum
		18	−78	−36	117	4.29	4.06	0.015^#^	R Cerebellum

A cluster-defining threshold (CDT) of *p* = 0.001 (T = 3.21) and a cluster based FWE –corrected threshold of *p* = 0.05 was used. MNI coordinates, k (number of voxels in the cluster), T-value, Z scores and FWE-corrected *p* values are provided. SFG = superior frontal gyrus; STG = superior temporal gyrus. N1: auditory; N2: DLPFC/PCC; N4: caudate; N8: cerebellum

^#^ The non-survivor of Bonferroni correction for multiple group comparisons

### Altered intra-network FC of caudate in patients with schizophrenia

**Fig. 5:**
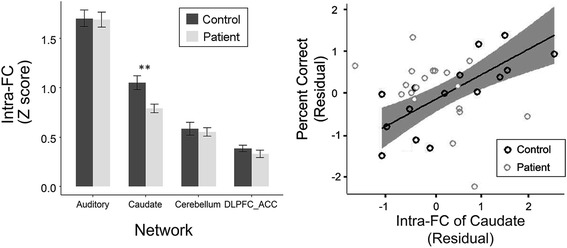
*Left panel:* The strength of intra-network functional connectivity in the caudate was significantly decreased in patients with schizophrenia compared to that in healthy controls. *Right panel:* Significant positive (Spearman) correlation occurred between the strength of intra-network FC of the caudate and percent correct of the button-press response in healthy controls, but not in patients with schizophrenia

Compared with healthy participants, patients with schizophrenia showed significantly decreased intra-network FC of the Caudate (*t* = 3.155, *p* = .003, Cohen’s d = 1.07 with 95% CI of 0.33 to 1.81; FDR corrected *p* = 0.012). Intra-network FC of other three networks, which had schizophrenia-altered spatial IC pattern, showed no significant difference between the two participant groups (left panel of Fig. 5). The results indicated that the strength of intra-network FC of the caudate for target-speech detection against informational speech masking was weaker in the patient participants than that in healthy controls.

### Correlation between strength of intra-network connectivity in the caudate and target-speech detection performance

A significantly positive correlation was revealed between the strength of intra-network FC (Z score) for the caudate and percent correct of target detection in healthy controls, but not in patient participants, with the PSS condition and the PSC condition combined (*r* = 0.624; *p* = 0.007; FDR corrected *p* = .026) (right panel of Fig. 5). No significant correlation was revealed for the other three networks. The results further confirmed that the speech-detection-related intra-caudate functional connectivity was normally underlying target speech detection against speech masking and impaired in patients with schizophrenia.

## Discussion

This study for the first time investigated schizophrenia-related changes in intra-network functional connectivity for target-speech detection against informational speech masking. The behavioral results showed that the speech-detection performance was poorer in the patient participants than their healthy controls under either the PSS condition or the PSC condition. Thus, under the informational speech masking condition, the reduced detection ability may at least partially account for the schizophrenia-induced speech-recognition impairment that have been previously reported [1, 3–7].

More importantly, the results of this study showed that compared to healthy controls, participants with schizophrenia exhibited significant decrease in both spatial covariation and strength of intra-network FC in the bilateral caudate. Also, in healthy controls, but not in patients with schizophrenia, the strength of intra-network FC in the bilateral caudate was positively correlated with the percent correct of target-speech detection. Thus, the weakness of speech-detection-related intra-caudate FC may be associated with reduced ability in target-speech detection against informational speech masking in patients with schizophrenia.

The caudate is part of extended language system with FC to the Broca’s and Wernicke’s areas [48], and is involved in speech inhibition and even more general response inhibition [49–52]. Particularly the caudate plays a role in accurate ambiguity resolution by regulating and monitoring the release of pre-formulated language segments for motor programming and semantic verification when the language processing cannot be entirely based on automatic mechanisms but needs recruiting controlled processes [50]. Thus, the results of this study suggest that in normal listeners the detection of target speech against informational speech masking may specifically involve both caudate-based suppression of disruptive masking signals and caudate-based regulating/monitoring the speech-motor programming and verification, leading to that the strength of intra-network connectivity in the caudate is positively correlated with the performance of target-speech detection.

In people with schizophrenia, both morphological and functional changes in the caudate have been reported [53–57]. In this study, participants with schizophrenia exhibited significantly decreased intra-network FC in the bilateral caudate under the target-speech-detection task. Moreover, the positive correlation between the strength of intra-network connectivity (Z score) for the bilateral caudate and the percent correct of target detection, occurred only in healthy controls. These results suggest that the schizophrenia-induced speech detection impairment under speech masking conditions can be accounted by dysfunction of the caudate.

It has been known that schizophrenia is associated with up-regulation of dopamine (DA) release in the caudate nucleus [58]. Also, schizophrenia-induced cognitive deficits (e.g., in working memory and attentional set shifting) are associated with functional deficits of the striatum [59]. Moreover, Meda et al. [60] have shown that the cingulate–thalamus–caudate component in fMRI ICA is associated with single nucleotide polymorphisms (SNPs) from dopamine transporter (DAT). Therefore, it would be of interest to know in future whether the schizophrenia-related functional impairment of the caudate is caused by schizophrenia-related changes in dopaminergic synapses in the caudate [54].

In addition to the caudate, this study also revealed changes in spatial covariation for the cerebellum and the STG in patient with schizophrenia. These brain regions are underlying some cognitive functions closely related to speech recognition under adverse listening conditions. For example, the bilateral STG is involved in not only the processing of target speech signals, but also the processing of the masking speech signals [9, 10]. Functional abnormalities of these brain regions in people with schizophrenia have also been reported previously [55, 61–65]. The increased spatial covariation pattern, but no increased intra-network FC of the STG and the cerebellum in patients with schizophrenia, suggests that in a target-speech detection task, the spatial covariation pattern of these brain networks is altered, but the mean strength of FC within networks remains intact.

## Conclusions

This study suggests that the caudate normally underlies detection of speech against informational speech masking. In people with schizophrenia the poor speech-detection performance against speech masking may be associated with a reduction of intra-network functional connectivity in the caudate, probably due to both the reduced suppression of masking signals and the reduced regulation of speech-motor processing.

## Abbreviations

ACC: anterior cingulate cortex
DAT: dopamine transporter
DLPFC: dorsal lateral prefrontal cortex
ECT: electroconvulsive therapy
HRTFs: head-related transfer functions
ICA: independent component analyses
mPFC: medial prefrontal cortex
OrbPFC: orbital prefrontal cortex
PCC: posterior cingulate cortex
PSC: perceived spatial co-location
PSS: perceived spatial separation
SCID-DSM-IV: Structured Clinical Interview for DSM-IV
SMR: signal-to-masker ratio
SNPs: single nucleotide polymorphisms
SPL: superior parietal lobule
